# Economic evaluation of the screening for Alzheimer’s disease in China

**DOI:** 10.3389/fnagi.2022.968842

**Published:** 2022-09-28

**Authors:** Yinan Ren, Dachuang Zhou, Qian Xing, Fangfang Gong, Wenxi Tang

**Affiliations:** ^1^School of International Pharmaceutical Business, China Pharmaceutical University, Nanjing, China; ^2^Center for Pharmacoeconomics and Outcomes Research of China Pharmaceutical University, Nanjing, China; ^3^Department of Hospital Group Office, Shenzhen Luohu Hospital Group Luohu People’s Hospital (The Third Affiliated Hospital of Shenzhen University), Shenzhen, China

**Keywords:** mild cognitive impairment, quality-adjusted life-year, incremental cost-effectiveness ratio, net monetary benefit, mini-mental state examination

## Abstract

**Background:**

We evaluated the cost-effectiveness of the screening in mainland China for Alzheimer’s disease (AD) patients aged over 60.

**Methods:**

Individuals in mainland China, received an initial screening by questionnaire on mental state, and those with scores deemed suspicious for AD were referred to diagnostic tests. A 9-state Markov model was developed to simulate the disease progression of a cohort of 100,000 subjects aging from 60 to 80. In addition, several scenarios were analyzed to assess the robustness under different screening frequency, starting age, the duration of drug effects, and the health status of subjects.

**Results:**

The ICER of AD screening was 26413.77 USD per QALY [quality-adjusted life-year (QALY)] compared with no screening. The number of deaths and severe AD cases who did not receive treatment averted by screening accounted for 0.076 and 0.006% of the total population, respectively, and the net monetary benefit was 128.29 USD per capita. Under the thresholds of one and three times the gross domestic product per capita, the probability of screening being cost-effective was approximately 18 and 77%, respectively. The ICER decreased to 18132.96USD per QALY when the drug effect was prolonged, and increased when the frequency of screening was increased, the starting age was postponed, and patients suffering from comorbidities were more. However, the number of severe AD cases and deaths declined when the screening frequency increased.

**Conclusion:**

Screening for AD in individuals over 60 can reduce the numbers of severe AD cases and deaths and may be cost-effective, depending on factors such as screening frequency, starting age of screening, and duration of drug effects. Additionally, mild cognitive impairment (MCI) is an important stage at which the burden of progression to AD may be reduced and the cost-effectiveness of screening may be improved.

## Introduction

Almost a fifth (18.70% in 2020) of China’s population is aged over 60, and 13.50 are over 65 (National Bureau of Statistics, 2021). There are 15.07 million cases of dementia among the senior individuals (≥60 years) in China, with 9.83 million of those (65.23%) diagnosed as Alzheimer’s disease (AD) (Zhou M. et al., 2019; Zhang et al., 2020), making China the country with the highest number of AD patients worldwide (Ferri et al., 2005; Ballard et al., 2011). In addition, 15.5% of the old adults suffer from mild cognitive impairment (MCI) (Jia et al., 2020). The annual costs of treating AD in China reached 167.74 billion USD in 2015 and are expected to exceed 1,887.18 billion USD by 2050 (Jia et al., 2018).

AD is a progressive neurodegenerative disorder that includes cognitive dysfunction, executive dysfunction, and personality and behavior changes. As AD worsens, patients gradually lose the ability to communicate, bowel and bladder control decreases, and the loss of the ability to swallow causes malnutrition and increases the risk of aspiration. In patients with AD, the risk of dying from infections, pressure sores, and pneumonia is greater than in healthy individuals (Boccardi et al., 2018). In 2019, the number of deaths from AD-related causes in China accounted for 19.8% of AD-related deaths worldwide, and the age-standardized mortality was 23.3 out of 100,000, slightly higher than the global average (Rujing et al., 2021). Moreover, the incubation period of AD is long, originally presenting as MCI in memory and thinking and the appearance of AD biomarkers in the brain. One study reported that 32% of patients with MCI over the age of 65 progressed to dementia within 5 years, while the other subset returned to normal cognitive function or remained stable. MCI should therefore be monitored to prevent AD-type dementia (Gatz et al., 2006).

Early diagnosis and intervention are effective ways to reduce the burden of AD. Potential promoters of AD, such as depression and vitamin resistance, can be detected and treated to improve the therapeutic outcome (Ballard et al., 2011). Early and accurate intervention may slow, halt, or prevent the progression or onset of AD and delay the associated morbidity (Chang and Silverman, 2004). At present, the diagnosis of AD mainly includes clinical evaluation, brain imaging, and laboratory findings. Clinical evaluation requires cognitive assessment, behavior assessment, and functional assessments, primarily using scales. Commonly used scales include the Mini-Mental State Examination (MMSE), the Montreal Cognitive Assessment, and Addenbrooke’s Cognitive Examination. Behavior assessment scales include the Neuropsychiatric Inventory and the Neuropsychiatric Inventory-Questionnaire. Common tools of functional assessment include the Instrumental Activities of Daily Living scale and Basic Activities of Daily Living scale. Brain imaging includes structural and functional imaging, and laboratory workups include cerebrospinal fluid tests and blood tests, which are mainly used to detect biomarkers of AD (Yifeng and Peiyuan, 2018; Jinzhou et al., 2021). A complete AD diagnosis should contain all these tests, but the associated costs are a public health concern worth evaluating.

An economic evaluation of opportunistic AD screening in South Korea was conducted using a Markov model in 2004; whether the screening was cost-effective depended on the severity of the disease, the treatment effect, the stage-specific treatment cost, age at screening, and the value of social willingness to pay (Yu et al., 2015). A static decision model constructed in 2014 to evaluate a one-time dementia screening program for the old individuals in the United Kingdom concluded that the program may be cost-effective and would be better with improvements in treatment and care (Dixon et al., 2015). However, there is still a gap in the field of AD screening in mainland China, and studies to date have focused on verifying the efficacy of diagnostic tools rather than evaluating AD screening as a public health service for the elderly (Yang et al., 2018; Yining et al., 2021).

Under this circumstance, the present study evaluated the effectiveness and cost-effectiveness of AD screening in mainland China, with a view to providing evidence for public health decision-making.

## Materials and methods

### Study design

The objective of this study was to assess the effectiveness of the AD screening program over no screening. Considering that patients with AD will gradually lose the capacity for self-care and therefore incur non-medical expenses, we conducted this study from a societal perspective.

The minimum age at screening and time horizon of the study were set from epidemiological findings in China and the characteristics of AD progression. First, the prevalence of AD in China increases in those over 40 and rises rapidly in individuals aged 70–74 (Rujing et al., 2021). Second, AD typically develops from mild to severe in six phases: β amyloid-positive, subjective cognitive decline, MCI, mild AD, moderate AD, and severe AD. The first three are termed preclinical stages. Approximately 3 years elapse from MCI to AD and 10 years from mild AD to severe AD (No authors listed., 2020; Autism spectrum disorder, 2021). MCI patients are at higher risk of developing AD than individuals with normal cognitive function, highlighting the importance of treating MCI to prevent progression to AD (Langa and Levine, 2014). We therefore set the age at which subjects entered the model at 60 years. The average life expectancy in China is 77.3 years, and therefore the simulation continued until the target population was 80 years old or died. The size of the cohort was 100,000.

The primary outcome indicator was the incremental cost-effectiveness ratio (ICER) and the secondary outcome indicators were the numbers of patients with severe and untreated AD that were avoided, the number of deaths avoided, and the net monetary benefit per capita. Net monetary benefit refers to the difference between the amount that the target population is willing to pay for the quality-adjusted life-year (QALY) gained and the cost incurred for it. The threshold used for the calculation of net monetary benefit was three times the GDP per capita in China in 2020 (34,106 USD) (Hongchao et al., 2020). Currency conversion was based on the 2021 exchange rate of 1 USD = 6.3725 CNY.

### Screening strategies

The strategy was a two-step screening, that is, using scale scores initially and providing diagnostic tests for individuals deemed positive for AD from the scale scores. All individuals aged > 60 years in mainland China were eligible to participate. The subjects and their insiders filled in the scale questionnaire under the guidance of general practitioners and received clinical diagnosis and examination by specialists in tertiary hospitals when the scale score indicated suspicion for AD.

The initial screening tool was the MMSE, the most widely used short cognitive test scale so far and the most commonly used assessment tool for dementia screening, cognitive grading, and outcome evaluation in clinical trials (Folstein et al., 1975; Tangalos et al., 1996; Lees et al., 2014; Tsoi et al., 2015). The diagnosis cut-off point was ≤ 22 for individuals lacking literacy, ≤ 23 for individuals with a primary school education, ≤ 24 for individuals with a secondary school education, and ≤ 26 for individuals with a university education.

### Model overview

Diagnostic tests included a physician’s examination, laboratory tests of bodily fluids (blood biochemistry, folic acid, serum vitamin B12, serum amylopsin, syphilis antibodies, thyroid function, and cerebrospinal fluid), and brain examination (magnetic resonance imaging and electroencephalography) (Jinzhou et al., 2021).

#### Natural history and Markov model

Screening was seen as taking actions on the disease early. The initial state of screening was equal to the natural state without screening. It was assumed that all patients diagnosed with AD by the screening would be treated. In addition, because the specificity of the screening tools is less than 100%, false positives may occur. This study assumed that false-positive subjects were treated for only 1 year and then considered to regain normal cognitive function. Given that the time interval between the symptoms being noticed and the diagnosis of AD was about 1.5 years (Rimmer et al., 2005), we also assumed that patients diagnosed through the screening were treated 2 years earlier than those who did not participate in the screening.

Both MCI and AD were included in the model. Subjects developed the disease following the sequence of normal cognitive function, MCI, and AD. AD was classified as mild, moderate, or severe according to the clinical dementia rating scale (Morris, 1993; Fuh et al., 2004). This scale grades the patients from responses to questions about six areas of cognitive function given by the patients themselves or their representatives. The scores are 0 (normal), 0.5 (problematic), 1 (mild injury), 2 (moderate injury), 3 (severe injury), 4 (critical injury), and 5 (fatal injury). In our study, a score of 1 was defined as mild AD, a score of 2 as moderate AD, and a score of 3–5 as severe AD.

**FIGURE 1:**
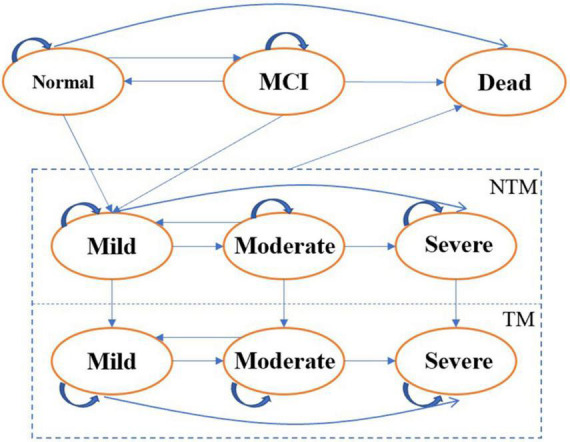
Markov model. NTM, not taking medicine; TM, taking medicine.

The model was constructed in Excel (Microsoft, Redmond, WA, USA) and the Markov states included normal cognitive function, MCI, mild AD, moderate AD, severe AD, and death. The four disease states were subdivided according to whether patients received treatment. The initial status was calculated by the prevalence of MCI and AD in the 60-year-old population, as well as by the proportion of mild, moderate, and severe AD patients. It was assumed that no one received treatment at the beginning. Figure 1 is a diagram of the model.

The length of the cycle in the model was 1 year. At the end of each cycle, subjects in each state could transit to the next state or die according to the transition probability. Drug therapy could reduce the transition probabilities (Neumann et al., 1999a; Fuh et al., 2004). At the same time, considering that there are currently no long-term clinical trials of related therapeutic drugs, we set the duration of drug effects at 3 years in the base case analysis, i.e., the transition probability of those who were treated was equal to that of patients who never received treatment (Burns et al., 2007; Homma et al., 2009; Yu et al., 2015; Birks and Harvey, 2018).

#### Model parameters

##### Clinical inputs

###### Prevalence and the proportion of Alzheimer’s disease patients in all stages

The prevalence of MCI was obtained from a systematic review of individuals over 60 in 22 of the 34 provinces in China. That study also stratified the findings by age, sex, and region. We used the age-specific and region-specific data (Xue et al., 2018). The prevalence of AD was derived from a meta-analysis of individuals over 55 in China, which pooled 51 studies and stratified the prevalence by age, geographical location, and other factors (Zhu et al., 2019). The proportion of AD patients was obtained from the “Investigation report on the diagnosis and treatment status of AD patients in China,” which is the first survey on AD diagnosis and treatment in 30 provinces and was released at the 13th Healthy China Forum on April 28, 2021 (Autism spectrum disorder, 2021).

###### Attribute parameters for screening and diagnosis

We included participation rate, the sensitivity and specificity of the MMSE, and diagnostic tests. Because of the lack of experience on relevant screening and the difficulty in obtaining real-world values, we set the participation rate at 80% (National Health Commission of the People’s Republic of China., 2020) according to the policies. The sensitivity and specificity of the MMSE were obtained from an economic evaluation of screening for AD in South Korea, and the sensitivity and specificity were 81.00 and 80.50%, respectively (Yu et al., 2015). For lack of data, the sensitivity and specificity of diagnostic tests were assumed to be 90% following an economic evaluation of screening for dementia in South Korea (Yu et al., 2015).

###### Transition probability

The incidence of MCI was used as the transition probability from normal cognitive function to MCI and derived from a cohort study of 16,921 healthy subjects over 55 years of age in four regions of China. The subjects were selected at random and followed for 4.5 years and the age-specific incidence of MCI was reported (Yuan et al., 2016). The transition probability of MCI patients was obtained from another cohort study in Shanghai, China, which followed 400 MCI patients for 3 years (Zhou B. et al., 2019). A study in Taiwan provided the annual transition probability of each AD stage from 365 patients with AD and an average follow-up period of 29 months. This study also measured the effect of cholinesterase inhibitors on the probabilities, but the transition probability from moderate AD to mild AD was not affected by the drug (Fuh et al., 2004).

###### Visit rate

The visit rate of patients with MCI was derived from the data of the neurology outpatient clinic of a general hospital in Chongqing province (Tingting, 2017). The visit rate of patients with AD (21.1%) and MCI (2.8%) were obtained from a national cross-sectional study including 46,011 subjects over 60 years old in China (Jia et al., 2020).

###### Mortality

Both age-specific natural mortality and disease-related mortality were included in the model. Disease-related mortality had been reported in the studies that detailed transition probabilities. The age-specific natural mortality was derived from the China Population and Employment Statistics Yearbook of 2017 (China Statistics Press, 2018).

###### Utility

The utility of healthy subjects and those who are died was set as 1 and 0, respectively. The utility of MCI originated from a cross-regional study in the United States that surveyed 679 patients in 13 regions using the Health Utility Index (HUI) mark 2 (Neumann et al., 1999b). And the utilities of AD patients derived from a survey among 416 subjects aged 65 years or older using EQ-5D in South Korea (Yu et al., 2015).

Supplementary Table 1 and Table 1 list the model parameters in detail.

**TABLE 1:** Age-specific natural mortality rate (China Statistics Press, 2018).

Age	Mortality
60–64	1.00%
65–69	1.63%
70–74	2.95%

##### Costs

Costs included direct medical costs, direct non-medical costs, indirect costs, intangible costs, and screening costs. Direct medical costs refer to medical service fees, examination fees, drug costs, and hospitalization costs. Direct non-medical costs include the costs of transportation, accommodation, meals, nutrition, healthcare equipment, and formal care. Indirect costs were defined as the loss of wages of patients and their caregivers and intangible costs included the anxiety and pain experienced by patients. Screening costs involved the costs of the project, scale measurement costs, and diagnostic examination fees. Direct costs, indirect costs, and intangible costs were obtained from an economic burden study conducted in 81 centers in 30 provinces in China, which reported the annual costs of treatment per capita according to the severity of AD (Yan et al., 2019). It was assumed that patients did not have comorbidities in base case studies. The costs incurred by patients who do not receive treatment refer to the indirect costs, nutrition costs, and healthcare equipment costs reported by a study on economic burden in China (Jia et al., 2018). The unit costs of the initial screening using the scales and the diagnostic tests were the median of the prices in all the provinces in mainland China or from expert opinion. The costs of items such as publicity and administration referred to other screening programs in China. The prices were inflated to 2021 prices using the CPI (Consumer Price Index) in China (National Bureau of Statistics of China, 2018) and were reported in United States dollars. Table 2 lists the cost parameters. The checklist and specific prices of diagnostic tests was shown in Supplementary Table 2, and the constitutions of costs of untreated patients were listed in Supplementary Table 3, while the CPI in each year were listed in Supplementary Table 4.

**TABLE 2:** Cost parameters.

Parameters	Value (USD)	Lower (USD)	Upper (USD)	Distribution	Resource
MCI, untreated	8.5	6.4	10.7	Gamma	Nuo et al., 2012
MCI, treated	8469.9	467.8	779.6	Gamma	
Mild AD, treated	12137.4	9103.4	15172.3	Gamma	Yan et al., 2019
Moderate AD, treated	12810.9	9608.2	16013.6	Gamma	
Severe AD, treated	22529.4	16897.0	28161.7	Gamma	
The proportion of direct medical costs	32.51%	−	−	−	Jia et al., 2018
The proportion of direct non-medical costs	15.62%	−	−	−	
The proportion of indirect costs	51.87%	−	−	−	
AD, untreated	13375.6	10031.7	23407.2	Gamma	Jia et al., 2018
False positive	155.5	116.7	272.2	Gamma	
Program	72185.2	54138.9	126324.0	Gamma	File
Scale per person	8.2	6.1	14.3	Gamma	File
Diagnostic test per person	152.4	114.3	266.7	Gamma	File calculation

### Sensitivity analysis

The uncertainty of the parameters was verified by sensitivity analyses, and the results of deterministic sensitivity analysis are presented as tornado figures. In probabilistic sensitivity analysis, a Monte Carlo simulation was conducted to draw from the distributions of all parameters randomly for 10,000 iterations, where the transition probabilities and utility followed the beta distribution and the costs followed the gamma distribution. Net monetary benefit was used to calculate the probability that the screening was cost-effective. The results of probabilistic sensitivity analysis are presented as scatter plots and cost-effectiveness acceptability curve.

### Scenario analysis

We conducted four scenario analyses. Scenario 1 used a screening frequency of 5 and 10 years. In scenario 2, the duration of drug effect was extended to life, i.e., the transition probability of patients receiving treatment would always be lower than that of patients who do not receive treatment. In scenario 3, we adjusted the starting age of screening to 65 and 70 years, and in scenario 4, patients could suffer from comorbidities. The costs of treatment were derived from the same studies used in the base case analysis. The distinction was that patients at this time may suffer from one to five comorbidities as well. We used the weighted average costs calculated according to the number of patients. Table 3 lists the parameters adjusted for each scenario.

**TABLE 3:** Scenario inputs.

	Parameter	Value	Lower	Upper	Distribution	Resource
Prevalence in Scenario3	MCI, 70∼80	18.46%	17.93%	18.99%	Beta	Xue et al., 2018
	AD, 65∼69	0.85%	0.60%	1.21%	Beta	Zhu et al., 2019
	AD, 70∼74	2.08%	1.61%	2.68%	Beta	
Scenario4	The cost of mild AD (USD)	15076.1	11307.1	18845.2	Beta	Yan et al., 2019
	The cost of moderate AD (USD)	18615.4	13961.5	23269.2	Beta	
	The cost of severe AD (USD)	28829.5	2163.5	36036.9	Beta	

## Results

### Base case analysis

The base case analysis showed that the costs increased by 289.44USD per person after screening was implemented, but the health benefits also increased by 0.011QALYs accordingly. That is, the additional cost of one unit of QALYs gained from screening was 26413.77USD, which was higher than the economic threshold of one time the GDP per capita (11,368.69 USD) but lower than three times the GDP per capita (34,106.08 USD). Compared with no screening, the number of deaths and severe AD cases who did not receive treatment avoided by screening accounted for 0.076 and 0.006 %of the total cases, respectively. The net monetary benefit was 128.29 USD per person per year. Tables 4, 5 detail the findings.

**TABLE 4:** Results of base case analysis.

	Costs (USD)	QALYs	Incremental costs (USD)	Incremental QALYs	ICER (USD)
Screening	24048.22	8.694	289.44	0.011	26413.77
No screening	23758.78	8.683			

**TABLE 5:** Secondary outcomes of the scenario analyses.

	Death avoid (n, %)	Untreated severe AD avoid (n, %)	NMB/USD
Base case	76 (0.076%)	6 (0.006%)	128.29
Every 5 years	1,080 (1.080%)	416 (0.416%)	−65.11
Every 10 years	431 (0.431%)	119 (0.119%)	−416.83
Drug effects	241 (0.241%)	1 (0.001%)	383.93
From65	105 (0.105%)	18 (0.018%)	45.10
From 70	264 (0.264%)	88 (0.088%)	−2685.34

### Sensitivity analysis

**FIGURE 2:**
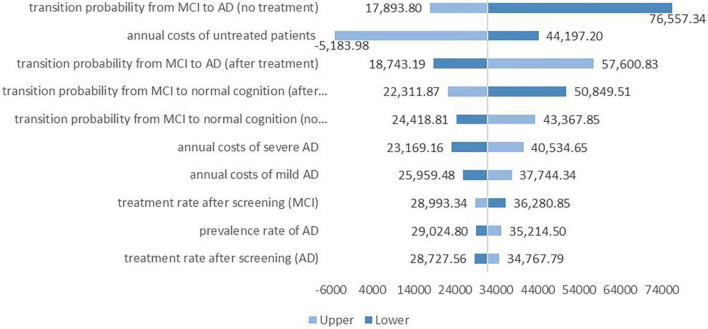
Deterministic sensitivity analysis.

Figure 2 depicts the results of the deterministic sensitivity analysis. Factors such as transition probabilities from MCI to AD and normal cognition no matter whether the patients would receive treatment, annual costs of untreated patients and expenses of patients of varying severity, treatment rate after screening and prevalence of AD influenced the cost-effectiveness of the screening greatly. The ICER was relatively low when the transition probability from MCI to AD before patients receiving treatment was high or when the transition probability from MCI to normal cognition after treatment was high, or when the treatment ratio of MCI patients after screening was high. Additionally, when the annual costs of untreated AD patients took the maximum value, the ICER of screening was negative.

**FIGURE 3:**
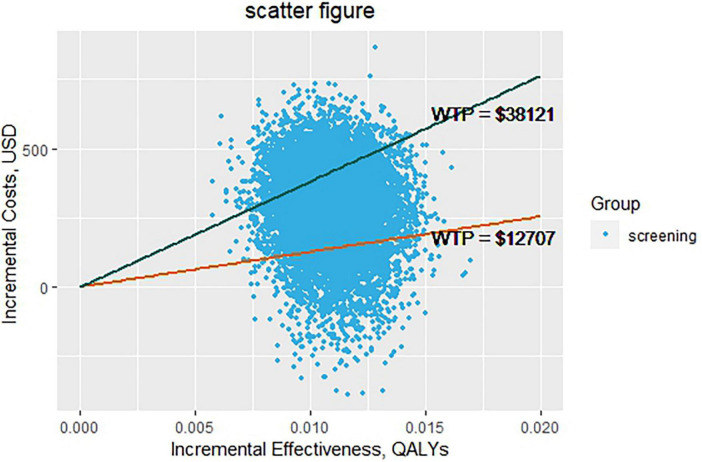
Probabilistic sensitivity analysis.

**FIGURE 4:**
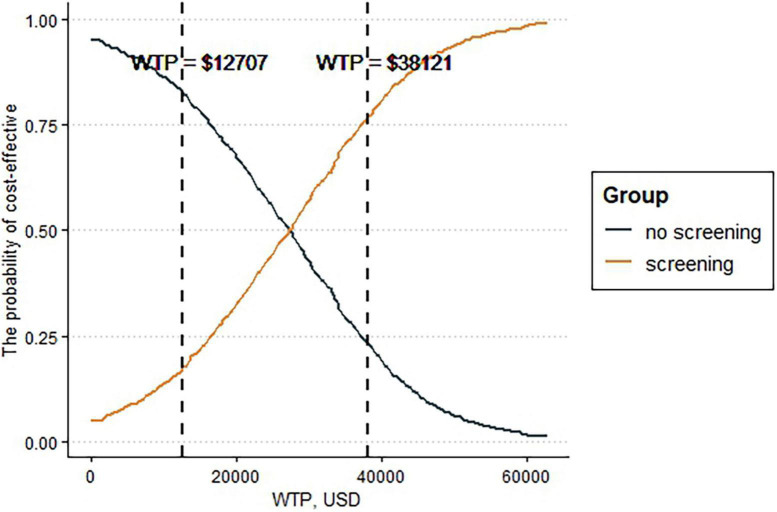
Cost-effectiveness acceptability curve.

Figures 3, 4 depict the results of the probabilistic sensitivity analysis. It could be found that 68% of the scatters were below the line of 3 times the GDP per capita. When the economic threshold was set at one time the GDP per capita, the probability of the screening being cost-effective was approximately 18%. When the threshold was raised to three times the GDP per capita, the probability increased to approximately 77%.

### Scenario analysis

Tables 5, 6 list the results of the scenario analysis. Increasing the frequency of screening was shown to improve the health benefits, with screening every 5 and 10 years increasing 0.040 and 0.021 QALYs, respectively. These two strategies would prevent some deaths (1.080 and 0.431%, respectively) and the number of patients with severe AD (0.416 and 0.119%, respectively) as well, but the ICER exceeded the threshold of three times the GDP per capita. When the duration of drug effect was extended, either the percentage of deaths avoided (0.241%) or the net monetary benefit per person (383.93 USD) increased, with the ICER decreasing to 18132.96 USD per QALY. When the starting age of screening was delayed, the number of deaths and severe AD cases remained lower than the numbers in the scenario without screening. However, the ICER exceeded the threshold of three times the GDP per capita. The health benefits did not change when patients had comorbidities, but the ICER increased significantly to 43173.63 USD per QALY, above the economic threshold of three times the GDP per capita.

**TABLE 6:** Results of scenario analyses.

	Costs (USD)	QALYs	Incremental costs (USD)	Incremental QALYs	ICER (USD)
**Scenario1 the frequency of screening**					
Every 5 years	25345.18	8.723	1586.40	0.040	39752.84
Every 10 years	24969.14	8.704	210.37	0.021	58145.74
No screening	23758.78	8.683			
**Scenario 2 the duration of drug effects**					
Screening	25782.84	8.730	348.29	0.019	18132.96
No screening	24434.55	8.711			
**Scenario3 starting age**					
From 65	20893.94	7.137	302.41	0.009	33174.27
No screening	20591.53	7.128			
From 70	19545.77	5.022	487.62	0.012	39408.72
No screening	19058.16	5.010			
**Scenario4 patients with complications**					
Screening	28063.66	8.694	473.09	0.011	43173.63
No screening	27590.56	8.683			

## Discussion

We found that the community screening program for AD in mainland China had the capacity to increase health benefits and reduce the incidence of severe AD and death. However, instead of saving costs as expected, the project increased the expenditures, possibly through the cost of screening itself and the higher expenses of treating the disease. Even so, AD screening remained cost-effective at a threshold of three times the GDP per capita, and the potential health benefits increased with frequent screening and longer duration of drug effect. Nonetheless, although the increase in the screening frequency appeared to favor health outcomes, the ICER increased accordingly, perhaps because once AD develops, it is almost impossible for patients to return to normal. In other words, the disease requires lifetime treatment. In addition, so far no drug has been identified that can halt the progression of AD (Lawlor et al., 2018; Craft et al., 2020; Koch et al., 2020; Kehoe et al., 2021). Therefore, a higher frequency of screening increases the costs of disease treatment. Accordingly, the degree of the impact of screening frequency on the medical expenses was greater than the degree of the impact on health benefits. The influence of drug efficacy on the results was consistent with our expectations; as more patients receive treatment, fewer cases progress to moderate or severe AD when the duration of therapy is extended to life. In this case, the quality of life could be improved and the costs of treatment could be reduced. In addition, we assumed that patients would be treated until the end of the simulation, while some studies proposed that patients would discontinue the treatment once they progressed to severe AD (Getsios et al., 2012). The ICER declined to 15600.18 USD per QALY after taking this assumption into consideration. This illustrates that improving the efficacy of the medicine favors the cost-effectiveness of the screening. We also found that the transition probabilities in the MCI stage exert a great impact on the results, further demonstrating the importance of the preclinical phase of AD. At the same time, an early starting age of screening is beneficial to the cost-effectiveness of the screening as well, perhaps because early screening can delay deterioration, further avoiding medical expenditures.

In addition, the cost-effectiveness of screening is affected by the components of the costs. First, comorbidities increase the direct medical costs, expanding the degree to which costs could reduce the cost-effectiveness of the screening, resulting in significant improvement of the ICER. Second, the high proportion of indirect costs and intangible costs favors the cost-effectiveness of the screening, and these costs account for approximately 51% of the economic burden of AD in China (Jia et al., 2018). This may be attributable to the psychological burden and wage loss incurred by both patients and caregivers whether patients receive medication or not. Therefore, screening reduces the costs incurred by patients without medication and reduces their probabilities of progression, thereby decreasing the cost required to obtain another unit of QALYs.

However, it should be noted that there are 34 provinces of various environments and lifestyles in China, so the prevalence of the disease and its economic burdens would vary by geographical region. The prevalence of MCI ranges from 2.4 to 27.8% (Qiu et al., 2003; Jiang et al., 2019; Deng et al., 2021), while the prevalence of AD fluctuates between 1.33 and 5.98% (Lü et al., 1998; Li et al., 2003). The annual cost per person fluctuates greatly between 2,384 and 19,144.36 USD (Wang et al., 2008; Xu et al., 2017; Jia et al., 2018). Therefore, the cost-effectiveness of implementing AD screening programs may vary by region.

The relevant studies cited focused mainly on the pathogenesis and treatment of AD, and evidence on the effectiveness and cost-effectiveness of AD screening is lacking. A few studies have looked at patients with dementia, without grouping them by the fundamental causes such as AD. The present study is an early investigation into the cost-effectiveness of AD screening projects in China, where the disease has a relatively complete clinical pathway and the study considers MCI status as well. In addition, the time horizon of the study is sufficiently long. Moreover, several scenarios were assessed to evaluate the impact of screening frequency, starting age, and comorbidities on the results. We also validated the importance of the preclinical status of AD in reducing the disease burden.

Some limitations should be noted. First, we had limited sources of published data related to AD screening, so the model was based on several assumptions and does not fully reflect real-world conditions. Second, the study was influenced to some extent by the quality of the second-hand data. For example, studies on the economic burden of AD in China differed in their findings, which may have affected our results. Additionally, the environmental and cultural differences between provinces require discussion on local screening strategies. Despite certain deficiencies, this study still provides evidence for public health decision-making in China and reference values for future studies.

## Conclusion

In China, the screening for AD in individuals aged over 60 can reduce the number of severe cases and deaths and may be a cost-effective choice. Improving the frequency of screening can increase the health benefits, but the cost-effectiveness of the screening should be discussed by threshold. Improving the efficacy of medical therapies for AD favors the cost-effectiveness of the screening programs. In addition, MCI is an important stage during which the disease burden can be reduced and the cost-effectiveness of screening can be improved.

## Data availability statement

The original contributions presented in this study are included in the article/Supplementary material, further inquiries can be directed to the corresponding author/s.

## Author contributions

YR made contribution to the conception and design of the study, the analysis and interpretation of the data, and the original writing and editing of the manuscript. DZ made contribution to the design of the study and the interpretation of the data. QX made contribution to the acquisition of the data and the review of the manuscript. WT made contribution to the design of the study and the review and editing of the manuscript. All authors also agreed both to be personally accountable for the author’s own contributions and to ensure that questions related to the accuracy or integrity of any part of the work and approved the submitted version.
