# Supplementary material for: Economic evaluation of the screening for Alzheimer’s disease in China
Source: Front Aging Neurosci. 2022 Sep 28;14:968842. doi: 10.3389/fnagi.2022.968842 (PMC9553991; doi:10.3389/fnagi.2022.968842)
Supplement: Supplementary file 1 [file Data_Sheet_1.docx]

Supplementary Table S1 Clinical inputs

|  | Parameters | Value | Lower | Upper | Distribution | Resource |
| --- | --- | --- | --- | --- | --- | --- |
| Prevalence | MCI, 60~70 | 9.93% | 9.59% | 10.23% | Beta | (32) |
|  | AD, 60~64 | 0.55% | 0.36% | 0.83% | Beta | (33) |
| Proportion | Mild AD | 48.53% | 36.40% | 60.66% | Beta | (18) |
|  | Moderate AD | 20.12% | 15.09% | 25.15% | Beta |  |
|  | Severe AD | 31.35% | 23.51% | 39.19% | Beta |  |
| Screening and Diagnosis | The participation rate of preliminary screening | 80.00% | 60.00% | 100.00% | Beta | Assumption |
|  | The participation rate of diagnostic test | 80.00% | 60.00% | 100.00% | Beta | Assumption |
|  | the sensitivity of the scales | 81.00% | 73.60% | 90.00% | Beta | (14) |
|  | the specificity of the scales | 80.50% | 72.50% | 88.60% | Beta |  |
|  | the sensitivity of diagnostic | 90.00% | 67.50% | 100.00% | Beta | (14) |
|  | the specificity of diagnostic | 90.00% | 67.50% | 100.00% | Beta |  |
| Treatment rate | AD | 21.10% | 15.83% | 26.38% | Beta | (6) |
|  | MCI | 2.80% | 2.10% | 3.50% | Beta | (6) |
|  | MCI-after screening | 80.00% | 60.00% | 100.00% | Beta | Assumption |
|  | AD-after screening | 80.00% | 60.00% | 100.00% | Beta |  |
| Transition probability | Normal to MCI | 8.09% | 6.04% | 10.83% | Beta | (55) |
|  | Normal to AD AD,60~64 | 1.12% | 0.60% | 1.94% | Beta | (36) |
|  | Normal to AD AD,65~69 | 1.62% | 0.94% | 2.60% | Beta |  |
|  |  |  |  |  |  |  |
|  | Normal to AD AD,70~74 | 5.96% | 4.30% | 8.06% | Beta |  |
|  | MCI to normal, untreated | 7.70% | 5.78% | 9.63% | Beta | (55) |
|  | MCI to mild AD, untreated | 5.40% | 4.05% | 6.75% | Beta |  |
|  | Mild to moderate, untreated | 24.60% | 22.14% | 27.06% | Beta | (27) |
|  | Mild to severe, untreated | 7.50% | 6.75% | 8.25% | Beta |  |
|  | Moderate to mild | 8.60% | 7.74% | 9.46% | Beta |  |
|  | Moderate to severe, untreated | 39.90% | 35.91% | 43.89% | Beta |  |
|  | MCI to normal, treated | 9.63% | 7.22% | 12.04% | Beta |  |
|  | MCI to mild AD, treated | 4.05% | 3.04% | 5.06% | Beta |  |
|  | Mild to moderate, treated | 21.90% | 19.71% | 24.09% | Beta |  |
|  | Mild to severe, treated | 2.20% | 1.98% | 2.42% | Beta |  |
|  | Moderate to severe, treated | 24.30% | 21.87% | 26.73% | Beta |  |
| Mortality | MCI | 0.48% | 0.36% | 0.59% | Beta | (55) |
|  | Mild AD, untreated | 2.80% | 2.52% | 3.08% | Beta | (27) |
|  | Mild AD, treated | 0.70% | 0.63% | 0.77% | Beta |  |
|  | Moderate AD, untreated | 12.00% | 10.80% | 13.20% | Beta |  |
|  | Moderate AD, treated | 1.50% | 1.35% | 1.65% | Beta |  |
|  | Severe AD, untreated | 20.90% | 18.81% | 22.99% | Beta |  |
|  | Severe AD, treated | 0.00% | 0.00% | 0.00% | - |  |
| Utility | Normal | 1 | 1 | 1 | - | - |
|  | MCI | 0.73 | 0.66 | 0.80 | Beta | (14,41) |
|  | Mild AD | 0.48 | 0.36 | 0.60 | Beta |  |
|  | Moderate AD | 0.34 | 0.26 | 0.43 | Beta |  |
|  | Severe AD | 0.10 | 0.08 | 0.13 | Beta |  |
|  | death | 0 | 0 | 0 | - | - |
| Discount | Discount rate | 5.00% | 0.00% | 8.00% |  | (21) |

Supplementary Table S2 Checklist of diagnostic test and unit price

| Inspection items | Value (USD) | Lower (USD) | Upper (USD) | Resource |
| --- | --- | --- | --- | --- |
| blood routine examination | 3.1 | 2.3 | 3.9 | The median price by provinces in mainland China in 2021 |
| Blood biochemistry | 27.3 | 20.5 | 34.1 |  |
| Thyroid function examination | 9.4 | 7.1 | 11.8 |  |
| Determination of folic acid | 3.5 | 2.6 | 4.4 |  |
| Determination of serum vitamin B12 | 4.7 | 3.5 | 5.9 |  |
| Determination of serum amyloid protein | 4.1 | 3.1 | 5.1 |  |
| Syphilis antibody determination | 3.1 | 2.3 | 3.9 |  |
| Routine examination of cerebrospinal fluid | 1.3 | 1.0 | 1.6 |  |
| electroencephalogram | 5.6 | 4.2 | 7.0 |  |
| MRI | 90.3 | 67.7 | 112.9 |  |
| Total cost | 152.4 | 114.3 | 190.5 |  |

Supplementary Table S3 Specific costs of untreated patients

| Items | Value (USD) | Lower (USD) | Upper (USD) | Resource |
| --- | --- | --- | --- | --- |
| Indirect costs | 65354.5 | 49015.9 | 81693.1 | (7) |
| Intangible costs | 5522.7 | 4142.0 | 6903.4 |  |
| Nutrition fee | 13254.5 | 9940.9 | 16568.2 |  |
| Equipment costs | 1104.0 | 828.0 | 1380.0 |  |
| Total costs | 85235.7 | 63926.8 | 106544.7 |  |

Supplementary Table S4 Consumer Price Index (CPI) in China (57)

| Year | CPI | Year | CPI |
| --- | --- | --- | --- |
| 2000 | 100 | 2011 | 103.4 |
| 2001 | 99.2 | 2012 | 102 |
| 2002 | 98.7 | 2013 | 101.3 |
| 2003 | 98.4 | 2014 | 101.3 |
| 2004 | 96.7 | 2015 | 102 |
| 2005 | 99.5 | 2016 | 104.1 |
| 2006 | 100.2 | 2017 | 105.4 |
| 2007 | 102.1 | 2018 | 104.3 |
| 2008 | 102.9 | 2019 | 103.9 |
| 2009 | 101.2 | 2020 | 101.8 |
| 2010 | 103.2 | 2021 | 102.7 |

References:

6. Jia L, Du Y, Chu L, Zhang Z, Li F, Lyu D, et al. Prevalence, risk factors, and management of dementia and mild cognitive impairment in adults aged 60 years or older in China: a cross-sectional study. Lancet Public Health. [Journal Article; Research Support, Non-U.S. Gov't]. 2020 2020-12-01;5(12):e661-71.

7. Jia J, Wei C, Chen S, Li F, Tang Y, Qin W, et al. The cost of Alzheimer's disease in China and re-estimation of costs worldwide. ALZHEIMERS DEMENT. [Journal Article; Multicenter Study; Observational Study; Research Support, Non-U.S. Gov't]. 2018 2018-04-01;14(4):483-91.

14. Yu SY, Lee TJ, Jang SH, Han JW, Kim TH, Kim KW. Cost-effectiveness of nationwide opportunistic screening program for dementia in South Korea. J ALZHEIMERS DIS. [Journal Article; Research Support, Non-U.S. Gov't]. 2015 2015-01-20;44(1):195-204.

18. Chinese ASD. Investigation report on the diagnosis and treatment status of Alzheimer's disease patients in China.; 2021.

21. Hongchao L, Guoen L, Shanlian H. hina Guidelines for Pharmacoeconomic Evaluations 2020. Beijing: China Market Press; 2020.

27. Fuh JL, Pwu RF, Wang SJ, Chen YH. Measuring Alzheimer's disease progression with transition probabilities in the Taiwanese population. Int J Geriatr Psychiatry. [Journal Article; Research Support, Non-U.S. Gov't]. 2004 2004-03-01;19(3):266-70.

32. Xue J, Li J, Liang J, Chen S. The Prevalence of Mild Cognitive Impairment in China: A Systematic Review. AGING DIS. [Journal Article; Review]. 2018 2018-08-01;9(4):706-15.

33. Zhu Y, Liu H, Lu XL, Zhang B, Weng W, Yang J, et al. Prevalence of dementia in the People's Republic of China from 1985 to 2015: a systematic review and meta-regression analysis. BMC PUBLIC HEALTH. [Journal Article; Meta-Analysis; Systematic Review]. 2019 2019-05-15;19(1):578.

35. Harvan JR, Cotter V. An evaluation of dementia screening in the primary care setting. J Am Acad Nurse Pract. [Journal Article; Review]. 2006 2006-08-01;18(8):351-60.

36. Yuan J, Zhang Z, Wen H, Hong X, Hong Z, Qu Q, et al. Incidence of dementia and subtypes: A cohort study in four regions in China. ALZHEIMERS DEMENT. [Journal Article; Research Support, Non-U.S. Gov't]. 2016 2016-03-01;12(3):262-71.

38. Tingting W. Research on the prevalence and community management strategies of mild cognitive impairment among the elderly in three districts of Chongqinng [dissertation]: Chongqing Medical University; 2017.

39. Jixing W, Zhenxing Z. Investigation on health care status of dementia patients over 55 years old in in urban and rural areas of Beijing. Chinese Journal of Rehabilitation. 2005(03):158-60.

41. Neumann PJ, Kuntz KM, Leon J, Araki SS, Hermann RC, Hsu MA, et al. Health utilities in Alzheimer's disease: a cross-sectional study of patients and caregivers. MED CARE. [Journal Article; Research Support, Non-U.S. Gov't]. 1999 1999-01-01;37(1):27-32.

55. Nuo W, Zhanjun Z, Dong C. Cost-effectiveness analysis of herbal interventional treatment using Markov model. China Journal of Chinese Materia Medica. 2012;37(18):2698-701.

57. China Statistical Yearbook.: China Statistics Press. http://www.stats.gov.cn/tjsj/ndsj/2021/indexch.htm
